# IoT-based health monitoring and social welfare access for Thailand’s older adults

**DOI:** 10.3389/fdgth.2026.1696118

**Published:** 2026-04-07

**Authors:** Chaturapron Chokphukhiao, Wonn Shweyi Thet Tun, Poomin Duankhan, Sakaowrat Masa, Patcharee Hongthong, Cholatip Pongskul, Somporn Chaiayuth, Jugsun Loeiyood, Piyathida Kuhirunyaratn, Bangonsri Jindawong, Nipitphon Seeooppalat, Sirapat Chiewchanwattana, Rina Patramanon, Khamron Sunat

**Affiliations:** 1 Information Technology International Program, College of Computing, Khon Kaen University, Khon Kaen, Thailand; 2 Center of Excellence in Coding Manpower Development and Digital Citizenship(CMDDC), Faculty of Education, Khon Kaen University, Khon Kaen, Thailand; 3 Digital Health Technology Unit, Khon Kaen University National Phenome Institute (KKUNPhI), Khon Kaen University, Khon Kaen, Thailand; 4 Department of System Biosciences and Computational Medicine, Faculty of Medicine, Khon Kaen University, Khon Kaen, Thailand; 5 College of Computing, Khon Kaen University, Khon Kaen, Thailand; 6 Department of Integrated Science, Faculty of Science, Khon Kaen University, Khon Kaen, Thailand; 7 Faculty of Business Administration and Accountancy, Khon Kaen Business School, Khon Kaen University, Khon Kaen, Thailand; 8 Department of Medicine, Faculty of Medicine, Khon Kaen University, Khon Kaen, Thailand; 9 Division of Public Health and Environment Service, Office of Public Health and Environment, Khon Kaen Municipality, Khon Kaen, Thailand; 10 Division of Information and Communication Technology, Khon Kaen Provincial Health Office, Khon Kaen, Thailand; 11 Department of Community, Family and Occupational Medicine, Faculty of Medicine, Khon Kaen University, Khon Kaen, Thailand; 12 Department of Biochemistry, Faculty of Science, Khon Kaen University, Khon Kaen, Thailand

**Keywords:** digital divide, digital health technology, IoT-based healthcare, older adults, social welfare access

## Abstract

**Introduction:**

Thailand's population is shifting, with 20% aged 60 and over in 2022, leading to healthcare and social welfare challenges. Digital technologies, particularly those using IoT, may improve health outcomes for older adults but face hurdles due to low digital literacy and a digital divide between urban and rural areas. This study investigated the accessibility and obstacles older adults encounter with social welfare services while assessing the effectiveness of current welfare programs.

**Methods:**

Data was gathered from 2,005 older adults through quantitative surveys regarding demographics, welfare accessibility, and the digital divide. A 6-month pilot program involving 80 users tested the daily usage of KATI smartwatches, WBP202 blood pressure monitors, and Contour Plus ELITE glucometers. All qualitative interviews, satisfaction ratings, and TAM data were directly supplied by the 80 older adult participants with the assistance of interviewers. Advanced features (alerts, SOS, predictive analytics, welfare integration) are planned for future scale-up.

**Results:**

Among the 2,005 respondents (mean age 68.9 years, 76.6% female), 64.2% had a monthly income of less than 5,000 THB, and 92.0% received the universal elderly allowance. In the pilot subgroup (*n* = 80), satisfactions were high (smartwatch 93.8%, blood pressure monitors 81.3%), and all TAM constructs exceeded 4.0 (range 4.259–4.373). Primary barriers to digital health technologies among older Thai adults include fear of online fraud (29.9%) and lack of digital equipment (15.8%).

**Conclusion:**

An IoT pilot showed good acceptability among smartphone users but risks increasing inequities without careful design for low-literacy, low-income, and homebound elders. Policies should focus on affordable devices, simple interfaces, and community training to promote inclusive digital aging.

## 1 Introduction

The growing elderly population in Thailand presents significant challenges for healthcare delivery and social welfare systems, highlighting the country's demographic transition toward an aging society (1, 2). By 2022, about 20% of Thailand's population is aged 60 or older, projected to rise to 35.8% by 2050, making it one of the fastest aging societies in Southeast Asia. In addition, United Nations population reports (3, 4) indicate that by 2050, one in six people worldwide will be over 65, with significant implications for healthcare systems internationally. Globally, demographic trends underscore a rapid rise in elderly populations, significantly impacting healthcare systems.

Traditional healthcare systems frequently fall short in delivering real-time health monitoring and personalized care, resulting in delayed medical interventions. Budget constraints and poor resource allocation exacerbate these issues. To address these shortcomings, innovative solutions like personal health budgets (PHBs) have been developed to enhance service integration and provide individualized care, especially for aging populations (5, 6). The intersection of digital technology and access to social welfare has become increasingly critical for addressing the needs of this growing elderly population (7, 8). Digital healthcare solutions encounter challenges tied to the “digital divide,” particularly affecting elderly populations. Barriers include limited technological literacy, physical impairments, economic constraints, and cultural resistance. In developing countries like Thailand, these issues are exacerbated by infrastructure limitations and disparities in internet connectivity and digital education. The World Health Organization's definition of healthy aging highlights the importance of mental, emotional, and social well-being, alongside physical health (9, 10). As Thailand continues to develop its welfare system and implement digital transformation, understanding how elderly citizens interact with and access services digitally becomes crucial to promoting healthy aging and social inclusion.

Internet of Things (IoT) technologies, especially wearable devices and sensors, enhance healthcare by providing real-time data, improving access to professionals, and aiding chronic disease management in the elderly. These health monitoring solutions enable continuous tracking of vital signs, facilitating early detection and proactive management of chronic conditions (11). A previous study by (12) conducted a comprehensive review of IoT healthcare interventions and revealed that successful implementation requires a robust data protection framework and culturally sensitive technology design. comprehensive user training programs. Moreover, IoT healthcare solutions provide significant advantages for the elderly, such as ongoing health monitoring, remote healthcare access, and tailored interventions. Advanced sensors facilitate real-time tracking of vital signs, allowing for early health risk detection and proactive measures (13). This integration of IoT devices in telemedicine can decrease emergency hospitalizations and enhance disease management for chronic conditions like diabetes, hypertension, and cardiovascular diseases, while also allowing immediate access to healthcare professionals (14). Additionally, telemedicine platforms that use IoT infrastructure improve healthcare access for underserved areas, especially rural regions with limited resources.

Machine learning algorithms analyze health data to provide personalized recommendations, medication reminders, and early warning systems for health deterioration (15, 16). Furthermore, artificial intelligence enhances digital healthcare solutions through data-driven insights and personalized recommendations, improving resource allocation and decision-making in budget-constrained systems. These technologies are beneficial for rural or underserved areas but require addressing barriers to digital exclusion among older adults to succeed in improving healthcare access and social inclusion (17, 18). Critical research gaps remain in IoT healthcare studies, particularly regarding the digital literacy barriers faced by elderly populations in low- and middle-income countries, despite promising technological advancements (19). Digital literacy is a crucial barrier for elderly populations in low- and middle-income countries, hindering the effective use of digital healthcare technologies (20). Longitudinal studies examining sustained health outcomes and quality-of-life improvements resulting from IoT interventions remain limited (21). Longitudinal studies on sustained health outcomes and quality-of-life improvements from IoT-based interventions are still limited (22). Implementing IoT healthcare solutions in Thailand presents unique challenges and opportunities. Previous studies in Southeast Asia indicate that IoT technologies can overcome healthcare infrastructure limitations and offer scalable solutions for the elderly care (23). The implementation of IoT healthcare technologies demands attention to data privacy, user-friendly design, and infrastructure compatibility. Ensuring robust encryption and regulatory compliance is vital for patient confidentiality and trust. Effective strategies must incorporate data protection frameworks, culturally sensitive designs, and user training to enhance technology adoption. Research indicates IoT's transformative potential in elderly healthcare, underscoring the importance of context-specific solutions that align with local infrastructure and cultural factors (24–26). IoT technologies offer potential for enhanced accessibility, personalization, and proactive solutions in healthcare, especially addressing the evolving needs of older adults.

Additionally, technology acceptance among older adults in low- and middle-income countries (LMICs) has been explored through both TAM and the Unified Theory of Acceptance and Use of Technology (UTAUT) (27). In Thailand, a 2025 meta-analysis highlighted that perceived usefulness and facilitating conditions are the strongest predictors of technology adoption, while effort expectancy is more significant than in high-income countries due to lower digital literacy and community support (28). The National Digital Government Development Plan (2023–2027) and the “No One Left Behind” elderly welfare portal aim to enhance digital services for older adults, but adoption is hindered by issues of digital literacy and trust (29). Evaluations of wearable health devices in aging populations in Southeast Asia and other LMICs indicate high satisfaction with simple devices supported by caregivers. However, barriers remain regarding cost, language, and fear of technology complexity (30, 31). This study extends existing evidence by analyzing actual device use and acceptance among a real-world cohort of elderly individuals in Thailand.

This study evaluates IoT-based health monitoring systems among elderly individuals in Thailand, analyzing their implementation across four provinces. It aims to assess the systems' efficacy in improving healthcare access and outcomes, as well as their role in facilitating access to social welfare services for older adults. Specifically, the study aimed to (1) examine how IoT devices are used for real-time monitoring of key health indicators such as blood pressure, blood glucose levels, and physical activity, evaluating the effectiveness of IoT devices for real-time monitoring of critical health indicators; (2) explore the role of IoT technology in facilitating access to social welfare services, including monthly allowances and routine health examinations, investigating the role of IoT in facilitating elderly access to social welfare services such as monthly allowances and routine health check-ups; (3) identify barriers to technology adoption among older adults, particularly in relation to digital literacy and concerns about security, by identifying and analyzing barriers to technology adoption, including digital literacy and security concerns; and (4) propose preliminary guidelines for provider-deployed IoT welfare models while highlighting the need for direct elderly acceptance studies.

## 2 Methods

### 2.1 Study design and sample collection

The research design employed a sequential exploratory mixed-methods approach (32), combining quantitative and qualitative data collection methods to assess the utility of IoT technologies in healthcare for older adults, with two separate participant groups. The study's quantitative phase included 2,005 older adults from four provinces, who completed standardized questionnaires on demographics, health, social welfare, and digital access. From these, 80 participants per province (Khon Kaen, Lampang, Phra Nakhon Si Ayutthaya, and Songkhla) will utilize IoT devices for six months, from January to June 2024.

**Figure 1:**
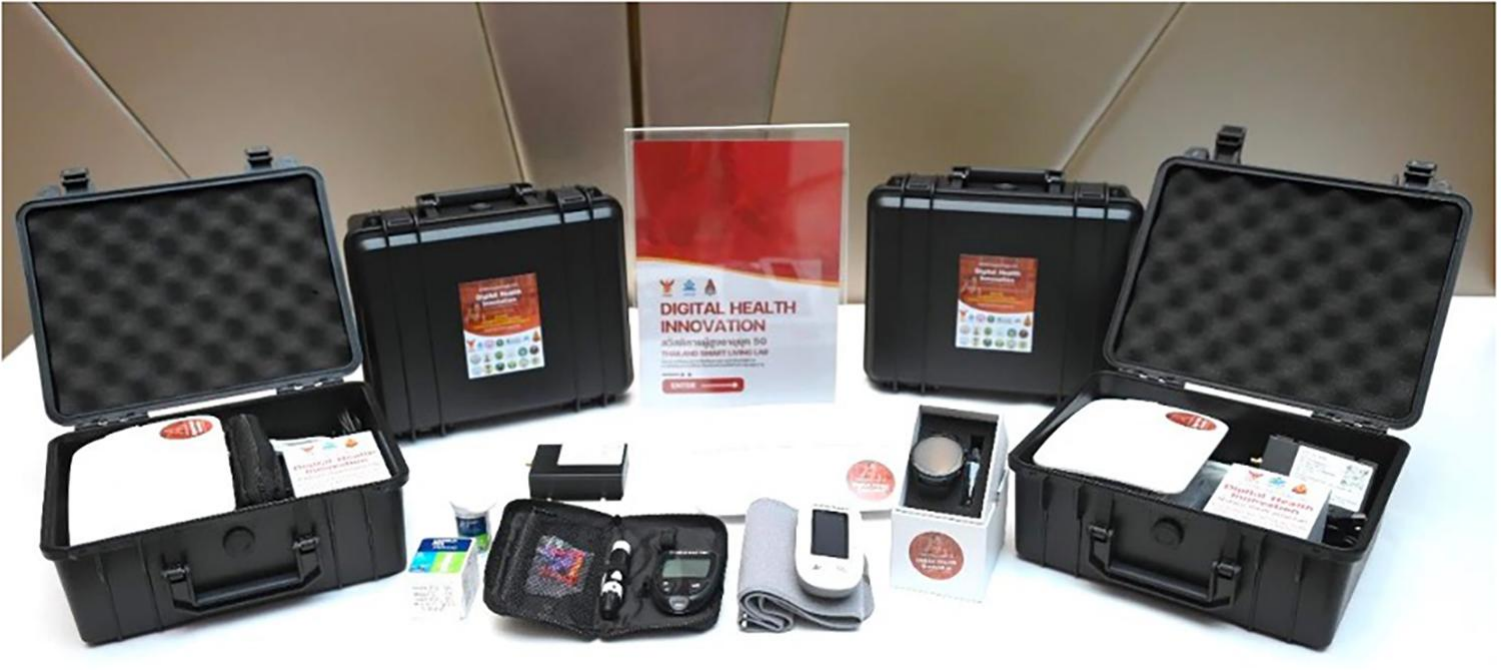
A set of equipment for digital services using IoT or 5G technology, including a KATI watch, a WBP202 electronic sphygmomanometer, a contour plus ELITE blood glucose monitor, and a medical data transmission machine (gateway) model MFC-AVA3.

The study involved 80 participants who monitored their blood pressure bi-daily with the WBP202 monitor and their blood glucose levels using the Contour Plus ELITE glucometer, as shown in Figure 1. Data were collected through the KATI smartwatch, which recorded various health metrics, including steps, calories, sleep, wrist temperature, pulse, arterial oxygen levels, and blood pressure, with all information being transmitted daily to an encrypted cloud storage system. During the initial setup, the Gateway device (MFC-AVA3) was installed by the research team by connecting it to a standard wall power socket located near the participant's bed or living room table. Upon activation, the Gateway automatically generated a Bluetooth hub that enabled automatic connectivity with the smartwatch, blood pressure cuff, and glucometer, eliminating the need for manual pairing or configuration by elderly participants. The study employed FDA-cleared, clinically validated devices: the WBP202 blood pressure monitor [validated per ISO 81060-2:2018 (33), mean differences 0.2/−0.9 mmHg systolic/diastolic], the Contour Plus ELITE glucometer [ISO 15197:2013 compliant, >98% accuracy within ±15 mg/dL/±15%; Freckmann et al. (34)], and the KATI smartwatch for continuous health metrics (steps, sleep, pulse rate, SpO2, temperature). All 80 participants underwent standardized 1-hour training covering device operation, measurement techniques, and hygiene protocols, with materials available in Thai and English. Blood pressure and glucose measurements were conducted twice daily, while smartwatch data was synced via Bluetooth every 5–15 min.

The 80 elderly pilot participants personally completed the device satisfaction ratings and the full 21-item Technology Acceptance Model (TAM) questionnaire through face-to-face, interviewer-assisted administration. Trained interviewers ensured comprehension by reading items aloud and providing neutral clarifications. Participants also engaged in in-depth interviews to share their experiences with the devices. Quantitative data was gathered through structured surveys measuring satisfaction with IoT devices and healthcare services, while qualitative data explored user experiences, challenges, and perceptions of the technology.

### 2.2 Selection criteria of the participants

**Figure 2:**
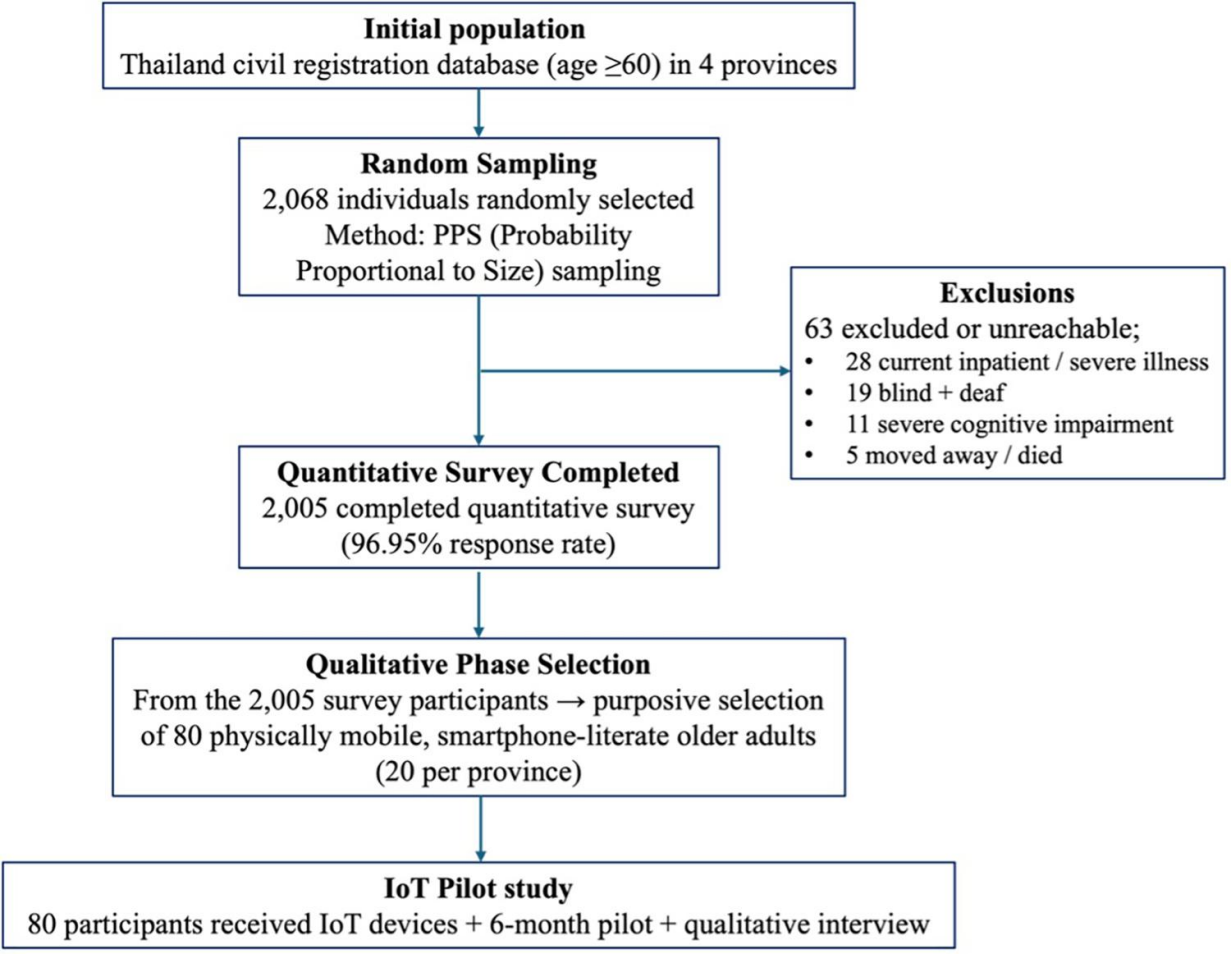
Study participant flow diagram of participants included in both the quantitative survey (*n* = 2,005) with a 96.9% response rate (2,005/2,068) and the IoT pilot (*n* = 80). Homebound, bedridden, and institutionalized older adults were not systematically targeted in the sampling frame.

The study focused on community-dwelling adults aged 60 and above in four provinces of Thailand: Khon Kaen, Lampang, Phra Nakhon Si Ayutthaya, and Songkhla, which represent different regions of the country. A two-stage probability proportional to size (PPS) sampling method was employed, selecting sub-districts based on the number of registered older adults, followed by a systematic random sample from updated civil registration lists. Field workers assessed individuals at home based on criteria including age (≥60 years), ability to communicate in Thai, and informed consent. Exclusions occurred for individuals who were blind and deaf simultaneously, currently undergoing inpatient treatment for severe illness, or had severe cognitive impairments. In cases of exclusion or refusal, a reserve list was used for substitution from the same village. Of the 2,068 individuals approached, 2,005 completed the questionnaire, yielding a response rate of 2,005/2,068 = 96.95%, as depicted in Figure 2. This high response rate was facilitated by official endorsement letters, the involvement of trusted village health volunteers, and in-person administration by trained interviewers who provided assistance as needed.

### 2.3 Implemented an IoT-based health monitoring system for older adults and planned future features

During the 6-month pilot, the 80 older adult participants (20 per province) actively used only the data-collection layer of the proposed three-tier system: KATI smartwatches for continuous monitoring, WBP202 blood pressure monitors, and Contour Plus ELITE glucometers (twice daily). Data were transmitted via Bluetooth to a smartphone and securely uploaded (AES-256/TLS encryption, PDPA-compliant) to a cloud platform, where participants could view their daily and historical data through a simplified mobile app. The satisfaction and technology acceptance results pertain only to the implemented components, while advanced features like real-time alerts and AI recommendations remain conceptual for future implementation.

### 2.4 Health monitoring system development

#### 2.4.1 System architecture

**Figure 3:**
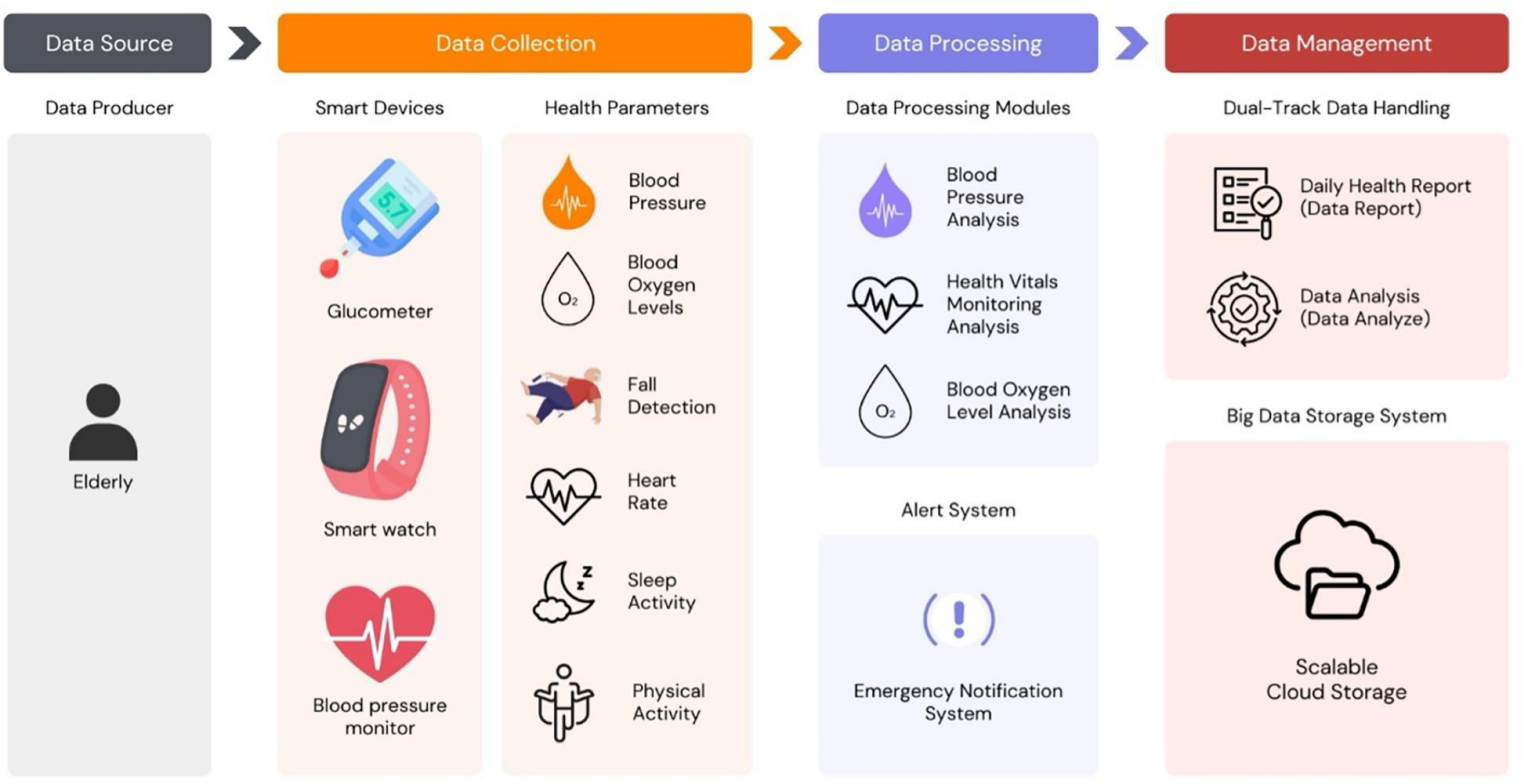
Conceptual three-tier IoT system architecture (data collection, processing, and management layers). Only the data-collection layer (KATI smartwatch, blood pressure monitor, glucometer) and basic cloud storage were implemented and used daily by 80 participants in the 6-month pilot; processing and management layers remain conceptual.

The proposed health monitoring system was designed via a three-tier IoT-based architecture, with each layer dedicated to distinct functionalities to ensure seamless, real-time monitoring and analysis of various health parameters. This architecture provides a scalable, reliable platform for data collection, processing, and management. Figure 3 illustrates the system's layered structure, comprising data collection, processing, and management.

#### 2.4.2 Data collection layer

The data collection layer is integral for obtaining health data from older patients via a network of smart devices, including a smartwatch for continuous monitoring of heart rate, activity, and sleep patterns; an oscillometric blood pressure monitor; and a glucometer for diabetes management. These devices track vital health parameters like blood pressure, blood oxygen levels (SpO₂), fall detection, heart rate abnormalities, sleep quality, and physical activity, all of which transmit data to a processing layer, creating a comprehensive health monitoring system tailored for older adults, as depicted in Figure 3.

#### 2.4.3 Data processing and data management layers

**Figure 4:**
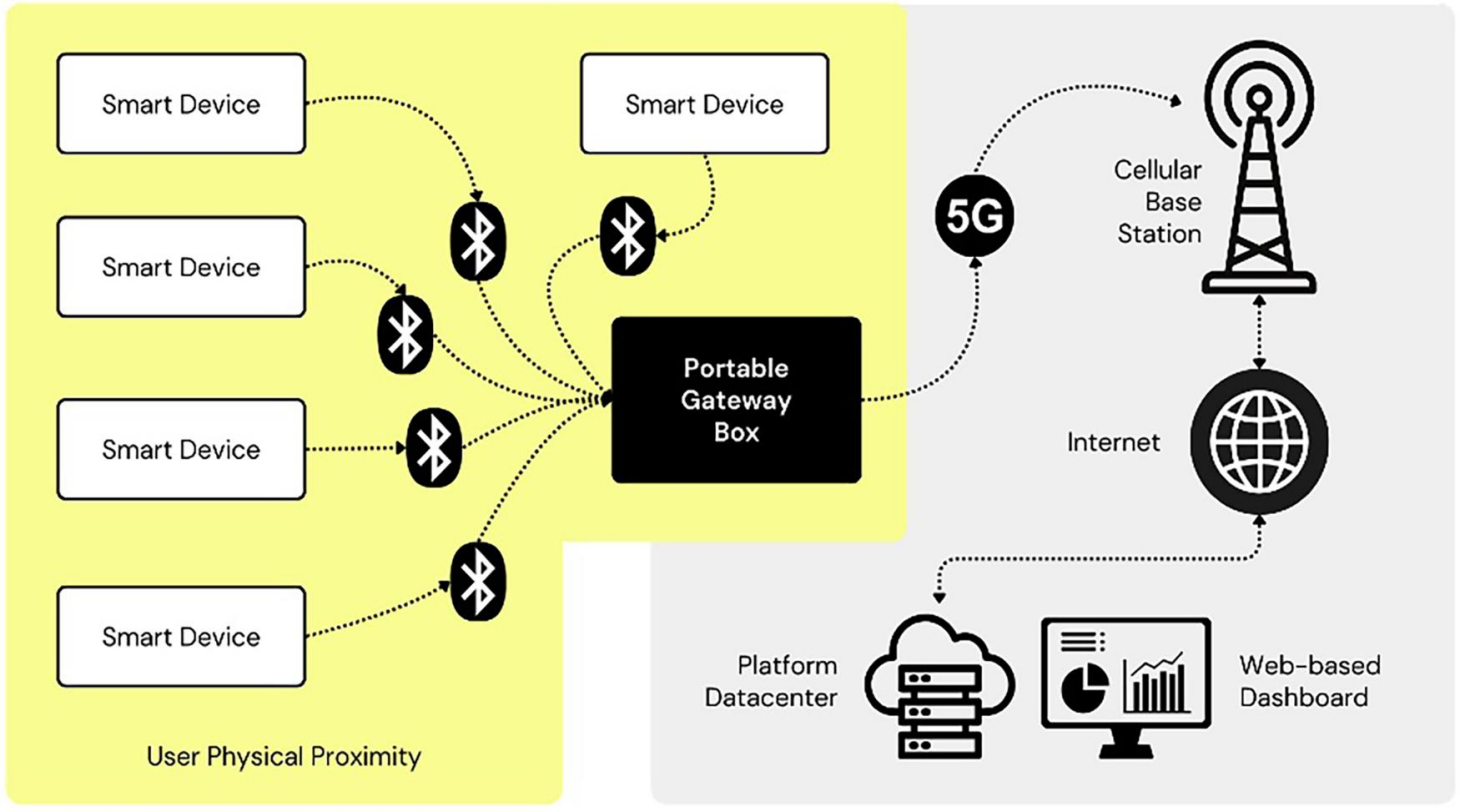
Envisioned IoT health-monitoring ecosystem. Only smart devices and smartphone-to-cloud upload were implemented in the 6-month pilot; all other components remain conceptual and planned for future scale-up.

The envisioned processing layer would be dedicated to real-time data analysis and alert generation, featuring specialized modules for blood pressure analysis that identify trends and deviations, comprehensive health vital monitoring that aggregates multi-sensor data, and continuous blood oxygen level evaluation for detecting respiratory issues, all working in conjunction with an emergency notification system that would trigger automated alerts when readings exceed predefined health thresholds. This system integrates with the data management layer, utilizing a dual-track approach for real-time and longitudinal data storage via daily health report generation and advanced data analysis (Figure 4). It features a scalable cloud-based big data storage system to maintain extensive health records, supporting longitudinal studies and personalized health analytics. This infrastructure facilitates insights into chronic conditions and long-term health trends while enabling routine monitoring and preventive healthcare through comprehensive historical data analysis.

### 2.5 Research instruments and data analysis

The qualitative analysis included a semi-structured in-depth interview guide to explore older adults' experiences with health issues, technology use concerns, access to welfare services, and views on IoT-based health monitoring. This guide was refined after review by three gerontology and digital health experts. The quantitative component was a structured questionnaire with nine sections (35): (1) general information, (2) health problems, (3) technology-related concerns, (4) demand for technology use, (5) technology usage patterns, (6) access to welfare services, (7) satisfaction with welfare services, (8) satisfaction with general technology use, and (9) satisfaction with health-monitoring technology (see supplementary Appendix A in the supplementary file).

Qualitative data from the 80 in-depth interviews were analyzed using thematic content analysis (36). Quantitative data analysis for the full survey sample (*n* = 2,005) focused on descriptive statistics, including frequencies, percentages, means, and standard deviations. In the IoT pilot subgroup (*n* = 80), these statistics were enhanced by Cronbach's *α* coefficients for the Technology Acceptance Model scales and Pearson's correlation coefficients to assess relationships among constructs such as Perceived Usefulness, Perceived Ease of Use, Attitude, and Behavioral Intention; see Supplementary Figure S1, Supplementary Tables S5, S6. Technology acceptance among the 80 IoT participants was evaluated using a validated Thai-language adaptation of the original Technology Acceptance Model (Davis) (37). The instrument consisted of 21 items rated on a 5-point Likert scale, addressing four constructs: Perceived Usefulness (PU, six items), Perceived Ease of Use (PEOU, six items), Attitude toward Using (AU, five items), and Behavioral Intention to Use (BI, four items). Device satisfaction was measured through three global questions for each device (KATI smartwatch, WBP202 blood pressure monitor, and Contour Plus ELITE glucometer) using a separate 5-point scale. Satisfaction rates reported the percentage of participants selecting either “satisfied” or “very satisfied.” Data analyses were conducted using IBM SPSS version 28 (38).

### 2.6 Mixed-methods sequential exploratory design

This study utilized a sequential, exploratory mixed-methods design to evaluate IoT-based health monitoring systems aimed at enhancing healthcare access and social welfare for older adults in Thailand. An analysis of the digital divide and health equity in four provinces involved a demographic survey (Table 3), informing the creation of a structured questionnaire administered to 2,005 older adults. Qualitative interviews with 80 IoT users and quantitative data on technology acceptance were analyzed (see section 3.3, Supplementary Tables S5, S6). This design effectively combined user insights with population-level trends, allowing for a comprehensive assessment of IoT effectiveness in an aging population, with integration of qualitative and quantitative data for a fuller understanding of the IoT's impact.

## 3 Results

### 3.1 Mixed-methods sequential exploratory results

#### 3.1.1 Demographic characteristics and living conditions of the older adult population

The study surveyed 2,005 older adults in four Thai provinces (Khon Kaen, Lampang, Phra Nakhon Si Ayutthaya, and Songkhla), revealing that 76.6% were female with a mean age of 68.9 years. Many participants faced financial difficulties: 48.6% had only primary education, 57.6% were unemployed, and 64.2% earned under 5,000 baht monthly (median income of 2,700 baht). Notably, 31.3% reported insufficient spending without debt, while 24.4% had sufficient spending but were in debt. Regarding living arrangements, 64.0% had dependents, 48.3% lived with spouses, 38.5% with grandchildren, and 14.8% lived alone. In caregiving, spouses primarily provided care (24.7%), but during illness, daughters took the lead (29.3%), followed by spouses (28.7%), as in Tables 1a, b.

**Table 1a:** Digital divide indicators and key sociodemographic characteristics by province and overall (*N* = 2,005; khon kaen *n* = 503, lampang *n* = 500, ayutthaya *n* = 501, songkhla *n* = 501).

Variable	Khon Kaen	Khon Kaen %	Lam-pang	Lam-pang %	Ayutthaya	Ayutthaya %	Songkhla	Songkhla %	Total	Total %
**1. Gender**										
Man	143	28.4	130	26	94	18.8	102	20.4	469	23.4
Female	360	71.6	370	74	407	81.2	399	79.6	1,536	76.6
**2. Age**										
60–69 years	279	55.5	286	57.2	297	59.3	309	61.7	1,171	58.4
70–79 years	192	38.2	180	36	168	33.5	179	35.7	719	35.9
80 years and up	32	6.4	34	6.8	36	7.2	13	2.6	115	5.7
Mean age [mean (SD), [min–max]]	[69.4 (6.4) 60–95]		[69.2 (6.2), 60–92]		[69.0 (6.0), 60–88]		[68.2 (5.5), 60–90]		[68.9 (6.0), 60–95]	
**3. Marital Status**										
Single	34	6.8	60	12	77	15.4	74	14.8	245	12.2
Legally married	280	55.7	263	52.6	217	43.3	247	49.3	1,007	50.2
Divorced/separated	16	3.2	43	8.6	39	7.8	33	6.6	131	6.5
Widowed	145	28.8	121	24.4	136	27.1	127	25.3	529	26.4
Living together without registration	28	5.6	13	2.6	32	6.4	20	4	93	4.6
**4. Education**										
Not educated	16	3.2	9	1.8	20	4	15	3	60	3
Primary education	314	62.4	168	33.2	290	57.9	204	40.7	974	48.6
Lower secondary school (Mathayom 3)	57	11.3	73	14.6	67	13.4	64	12.8	261	13
High School/Vocational Certificate/associate degree	86	17.1	150	30	83	16.6	113	22.6	432	21.5
Bachelor's degree and above	30	6	102	20.4	41	8.2	105	21	278	13.9
**5. Career**										
Not working	268	53.3	267	53.4	294	58.7	326	65.1	1,155	57.6
Retired civil servant	26	5.2	59	11.8	21	4.2	30	6	136	6.8
Trade	59	11.7	82	16.4	73	14.6	54	10.8	268	13.4
Farmer	67	13.3	20	4	2	0.4	19	3.8	108	5.4
General employee	47	9.3	51	10.2	67	13.4	48	9.6	213	10.6
Private business/Business owner	13	2.6	17	3.4	15	3	22	4.4	67	3.3
Owner of rental room/house for rent	0	0	1	0.2	1	0.2	1	0.2	3	0.1
Other (village health volunteers, etc.)	41	8.2	27	5.4	33	6.6	14	2.8	115	5.7
**6. Average Income/Allowance**										
Less than 5,000 baht	356	71.9	287	58.6	348	70.4	251	55.2	1,242	64.2
5,000–15,000 baht	68	17.8	106	21.6	106	21.5	126	27.7	426	22
More than 15,000 baht	51	10.3	97	19.8	40	8.1	78	17.1	266	13.8
Median income/allowance [median (IQR), min–max]	[2,200 (4,900), 600–61,000]		[3,600 (9,500), 600–1,00,600]		[2,600 (4,850), 600–55,000]		[4,300 (54,400), 600–1,01,700]		[2,700 (72,500), 600–1,01,700]	
**7. Life Satisfaction**										
Not enough to spend and in debt	150	30.3	105	21.4	122	24.7	94	20.7	471	24.4
Not enough to spend and not in debt	193	39	122	24.9	184	37.2	106	23.3	605	31.3
Enough to spend but nothing left to save	105	21.2	155	31.6	116	23.5	131	28.8	507	26.2
Enough to spend and able to save	47	9.5	108	22	72	14.6	124	27.3	351	18.1
**8. Care Obligations**										
No care obligations	148	29.4	224	44.8	179	35.7	171	34.1	722	36
Have care obligations (children/grandchildren/relatives)	355	70.6	276	55.2	322	64.3	330	65.9	1,283	64
**9. Living with family**										
Living alone	43	8.5	109	21.8	65	13	80	16	297	14.8
Spouse/husband/wife	282	56.1	246	49.2	198	39.5	242	48.3	968	48.3
Son	146	29	109	21.8	157	31.3	151	30.1	563	28.1
Daughter	203	40.4	106	21.2	153	30.5	161	32.1	623	31.1
Son-in-law/Daughter-in-law	71	14.1	61	12.2	49	9.8	52	10.4	233	11.6
Grandchildren	246	48.9	139	27.8	224	44.7	162	32.3	771	38.5
Relative	29	5.8	40	8	42	8.4	39	7.8	150	7.5
**10. Regular Caregiver information**										
No regular caregiver	148	29.4	224	44.8	179	35.7	171	34.1	722	36
Spouse/husband/wife	153	30.4	108	21.6	100	20	135	26.9	496	24.7
Son	100	19.9	63	12.6	109	21.8	125	25	397	19.8
Daughter	155	30.8	73	14.6	114	22.8	149	29.7	491	24.5
Son-in-law/Daughter-in-law	12	2.4	14	2.8	10	2	15	3	51	2.5
Grandchildren	38	7.6	31	6.2	77	15.4	66	13.2	212	10.6
Relative	6	1.2	29	5.8	21	4.2	24	4.8	80	4
**11. Caregiver During Illness**										
No caregiver during illness	75	14.9	126	25.2	154	30.7	106	21.2	461	23
Spouse/husband/wife	170	33.8	155	31	100	20	151	30.1	576	28.7
Son	121	24.1	88	17.6	116	23.2	154	30.7	479	23.9
Daughter	190	37.8	100	20	121	24.2	177	35.3	588	29.3
Son-in-law/Daughter-in-law	18	3.6	19	3.8	10	2	19	3.8	66	3.3
Grandchildren	46	9.1	38	7.6	83	16.6	87	17.4	254	12.7
Relative	8	1.6	39	7.8	26	5.2	31	6.2	104	5.2

All percentages are row percentages (within-province distribution).

**Table 1b:** Comparison of study sample characteristics with Thailand national elderly population data (NSO, 2024).

Characteristic	Study Sample (*n* = 2,005)	National NSO Data (2024 Survey)	Comparison
**Sex**			
Female	76.6%	58.0%	*Over-represented ↑*
Male	23.4%	42.0%	*Under-represented ↓*
**Age group**			
Mean age (SD)	68.9 (6.0) years	∼68–70 years (est.)	*Comparable*
60–69 years	58.4%	59.0%	*Comparable ≈*
70–79 years	35.9%	30.0%	*Slightly over-represented ↑*
80 years and above	5.7%	∼11.0%	*Under-represented ↓*
**Education level**			
No formal education/below primary	3.0%	67.2%	*Substantially under-represented ↓*
Primary education	48.6%	12.6%	*Over-represented ↑*
High school/vocational	21.5%	4.8%	*Over-represented ↑*
Bachelor's degree and above	13.9%	7.9%	*Over-represented ↑*
**Employment status**			
Not working/retired	57.6%	∼66.0%	*Comparable (slightly lower) ≈*
Still working	42.4%	∼34.0%	*Slightly over-represented ↑*
**Residential area**			
Urban	Not reported by province	∼34.2% (national urban population)	*Urban-rural mix across 4 provinces*

NSO national data sourced from the Thailand NSO Survey of Older Persons 2024 and NSO Statistical Yearbook 2024. The study sample comprised the older adults across 4 provinces (Khon Kaen, Lampang, Ayutthaya,and Songkhla). The overrepresentation of women, older age strata (70–79 years), and higher-education groups likely reflects community-level sampling and survivorship patterns. Discrepancies in education categories reflect differences in classification between the study questionnaire and NSO survey coding. ↑ = over-represented relative to national; ↓ = under-represented relative to national; ≈ = broadly comparable.

#### 3.1.2 Technology access and usage patterns among older adults

Unless explicitly stated otherwise, all findings on demographics, socioeconomic status, social-welfare access, digital divide, and barriers are derived from the full quantitative sample (*n* = 2,005). Findings on technology acceptance (TAM scores), device satisfaction, and user experience are derived exclusively from the 80 older adults who used the IoT devices for 6 months with the assistance of the interviewers. The proposed advanced system features, including alerts, predictive analytics, and welfare integration, remain unavailable for future development. The pilot participants rated their overall satisfaction with the three study-provided devices after 6 months of daily use. Satisfaction was very high for the KATI smartwatch (93.8% satisfied/very satisfied), high for the blood pressure monitor (81.3%), and high for the glucometer (78.8%) (Table 2). In addition, the satisfaction with the Gateway MFC-AVA3 data-transmission hub was good (70.0%). Most participants reported they rarely noticed or interacted with it after initial setup, which is the intended design for minimizing user burden. Strong technology adoption was noted, with smartwatches and blood pressure monitors showing high satisfaction rates; however, qualitative insights identified digital literacy as a barrier.

**Table 2:** Technology access and device satisfaction after 6 months of daily use of the study-provided IoT devices (*n* = 80 for each device; single global satisfaction item, 5-point scale).

Equipment	Little Satisfied	Little Satisfied %	Moderately Satisfied	Moderately Satisfied %	Most Satisfied	Most Satisfied %
KATI smartwatch	2	2.5	3	3.8	75	93.8
WP202 Blood pressure monitor	4	5.0	12	15.0	65	81.3
Contour Plus ELITE glucometer	3	3.8	14	17.5	63	78.8
Gateway MFC-AVA3 (data transmission hub)	3	3.8	21	26.3	56	70.0

#### 3.1.3 Provincial perspectives on digital health innovation and health equity through digital divide analysis

The study assessed smartwatch technology for health monitoring in older populations across four Thai provinces: Lampang, Khon Kaen, Phra Nakhon Si Ayutthaya, and Songkhla (Table 3). Significant digital divides influenced technology adoption and health equity. Khon Kaen faced high barriers, with 62.4% having only primary education and prevalent fraud concerns (29.9%). Lampang showed moderate digital divide effects, leading to better adoption and high health examination access (83.6%). Ayutthaya experienced significant educational and economic barriers (70.4% earning less than 5,000 baht), limiting technology satisfaction. Songkhla had the most favorable profile with moderate education levels and lower poverty rates (55.2% earning less than 5,000 baht).

**Table 3:** Digital divide indicators, socioeconomic gaps, and health equity implications by province and overall (*N* = 2,005).

Digital Divide Indicators by Province				
Province	Education Gap	Income/ allowance Gap	Digital Literacy Barriers	Welfare Access Rate
Khon Kaen	high primary only (62.4%) vs. low higher education (6%)	71.9% earn than 5,000 baht	High fraud concerns (29.9%)	Monthly allowance: 92%
Lampang	Moderate primary (33.2%) vs. high higher education (20.4%)	58.6% earn <5,000 baht	Moderate digital confidence	Health exams: 83.6%
Ayutthaya	High primary only (57.9%) vs. low higher education (8.2%)	70.4% earn than 5,000 baht	Cost concerns prominent	Variable service access
Songkhla	Moderate primary (40.7%) vs. high higher education (21%)	55.2% earn than 5,000 baht	Better technology acceptance	Strong welfare integration
**Digital Divide Dimensions**				
**Dimension**	**Advantaged Group**	**Disadvantaged Group**	**Gap Severity**	**Impact on Health Equity**
Access Divide	Smartphone users	No smartphone/basic phone users	Moderate	Limits real-time health monitoring
Skills Divide	Higher education (13.9%)	Primary education only (48.6%)	High	Prevents effective technology use
Economic Divide	Income >15,000 baht (13.8%)	Income <5,000 baht (64.2%)	Severe	Creates technology affordability barrier
Usage Divide	Active, mobile elderly	Homebound/bedridden elderly	Critical	Excludes most vulnerable populations
Geographic Divide	Urban areas (Lampang/Songkhla)	Rural areas (Khon Kaen/Ayutthaya)	Moderate-High	Unequal infrastructure and support
**Health Equity Impact Assessment**				
**Health Service**	**Current Access Rate**	**Digital Enhancement**	**Equity Risk**	**Vulnerable Group Impact**
Monthly Allowances	92%	High digital integration	Low-Medium	May exclude non-tech users
Health Monitoring	83.6% (annual exams)	IoT real-time tracking	High	Benefits tech users only
Emergency Services	Variable	SOS smartwatch feature	High	Requires device ownership
Medication Management	Not specified	App-based reminders	Medium	Literacy-dependent
Specialist Consultation	Limited	Telemedicine integration	High	Widens urban-rural gap

The lower panels summarize the five dimensions of the digital divide and a qualitative health-equity risk assessment based on study findings.

The analysis of the digital divide highlighted five key factors impacting health equity, as shown in Table 3. First, the access divide showed a moderate gap with the elderly who owned smartphones, compared to those who did not. Second, the skills divide revealed a significant disparity, as individuals with higher education (13.9%) are far more likely to have digital skills than those with only primary education (48.6%). Third, the economic divide was severe, with 64.2% of those earning less than 5,000 baht facing greater barriers to digital access than just 13.8% of those earning over 15,000 baht. Fourth, the usage of underscores is a critical gap between active mobile elderly users and those who are homebound or bedridden, limiting their ability to engage with digital tools. Finally, the geographic divide was moderate to high, with urban residents having better access to digital resources than those in rural areas.

Concerns about equitable digital access for health show significant disparities. While 92% access is achieved through monthly allowances, real-time IoT tracking poses equity risks, primarily benefiting tech users. Emergency services via SOS smartwatches and telemedicine may worsen urban-rural gaps due to ownership requirements. Over 64.2% of older people earning less than 5,000 baht struggle to obtain technology, affecting vulnerable homebound groups. Without addressing the digital divide, local contexts, prices, and inclusive integration, digital health technologies could exacerbate health inequities, despite their potential to enhance healthcare access for older adults. Detailed results regarding social welfare access, utilization, and needs for older adults' quality of life are presented in Supplementary Tables S1–S4 of the supplementary information.

### 3.2 Technology acceptance analysis

The correlation analysis and reliability assessment of 21 variables across four constructs—Perceived Usefulness (PU), Perceived Ease of Use (PEOU), Attitude (AU), and Behavioral Intention (BI)—indicated significant relationships and reliability patterns. Strong internal correlations were observed, particularly between PU4 and PU6 (*r* = 0.713) and PEOU5 and PEOU6 (*r* = 0.744), evidencing good convergent validity (see Supplementary Figure S1). Notably high correlations like AU4-AU5 (*r* = 0.793) suggest potential redundancy. Reliability analysis via Cronbach's Alpha revealed excellent internal consistency for PU (*α* = 0.938) and outstanding reliability for BI (*α* = 0.989), while PEOU showed good reliability (*α* = 0.836). The AU construct showed questionable reliability (*α* = 0.664), below the acceptable 0.7 threshold, and thus requires further refinement. The overall questionnaire showed good reliability (*α* = 0.857), as shown in Supplementary Table S5.

The evaluation of acceptance levels for digital health equipment indicated strong positive responses: Perceived Usefulness (PU) averaged 4.354, Perceived Ease of Use (PEOU) averaged 4.373, Attitude Towards Use (AU) averaged 4.352, and Behavioral Intention (BI) averaged 4.259, all exceeding the 4.0 acceptance threshold (Supplementary Table S6). The low standard deviations suggest consistent agreement among respondents, which aligns with common interpretations and mean scores above 4.0 on a 5-point Likert scale (where 5 = strongly agree) in Technology Acceptance Model (TAM) studies within healthcare contexts [Davis et al. (37); Garavand et al. (39); Tao et al. (40)]. The study demonstrates strong acceptance of various digital health equipment by elderly users.

### 3.3 Problems and obstacles in conducting research

There were several limitations in this study, as listed in Table 4. This study identified several limitations, notably a sample group of older adults able to travel for interviews, excluding homebound individuals, which may overlook the most vulnerable elderly population. Key health issues such as vision, sleep, and memory problems should be considered in welfare rights applications. Additionally, 48.6% of older adults with only primary education faced challenges with digital skills. Device affordability hindered 64.2% of low-income elderly from accessing the internet, while 15.2% in rural areas reported limited internet access. Security concerns like online fraud affected 29.9% of users, and complex language in interfaces presented barriers for those with lower education levels. The study was also restricted in geographic scope, participant diversity, and group adaptability.

**Table 4:** Technology adoption barriers and facilitators.

Factor	Barrier Level	Affected Population	Mitigation Strategy	Implementation Status
Device Cost	High	64.2% low-income elderly	Subsidized device programs	Not implemented
Digital Literacy	High	48.6% primary education only	Gradual training programs	Partially planned
Internet Access	Moderate	Rural areas (15.2% limited coverage)	Infrastructure development	In progress
Security Concerns	High	29.9% fear online fraud	Trust-building education	Partially planned
Physical Limitations	Critical	Excluded from study	Adaptive technology design	Not addressed
Language/Interface	Moderate	Lower education groups	Simplified, local language UI	Partially implemented

Several steps are proposed to address technology access issues for the elderly: gradual training programs tailored to various skill levels, education to build trust in technology's benefits, subsidized device programs for low-income seniors, simpler interfaces in local languages, and employing tech-savvy elderly as community role models (Table 4). Infrastructure development for rural internet access is advancing, targeting moderate barriers, yet adaptive technology for the elderly remains lacking. Training for working groups is essential for providing practical guidance, considering geographical disparities, and the varied technological capacities of different elderly groups.

### 3.4 Policy recommendations to enhance access to social welfare rights for older adults

The Social Welfare Rights Enhancement Program offers extensive support for older adults and disadvantaged citizens via collaborations among 24 agencies, including ministries and educational institutions. It addresses health, economic aid, and environmental aspects, utilizing television, community networks, and digital applications for information dissemination. The program, part of the Thailand Smart Living Lab, emphasizes digital health innovations and medical IoT, achieving over 80% user satisfaction in Lampang and Khon Kaen. Funding includes 20% for 5G infrastructure from the NBTC and Ministry of Digital Economy, 15% for digital research from the Ministry of Higher Education, and 25% from various health and social budgets through a partnership model with a 20:30:50 ratio among government, private, and social entities, focusing on infrastructure, smart technologies, and health information management.

## 4 Discussions

Our study examined the integration of IoT technologies in healthcare for older adults in Thailand, with an emphasis on technology adoption and access to social welfare. The sample consisted mostly of women (76.6%) aged 60–69, reflecting aging trends in Southeast Asia as noted by Knodel & Chayovan (41). Most participants (64.2%) reported monthly allowance/support of less than 5,000 baht (∼146 USD), with a median of 2,700 baht. Rather than reflecting earned income, these figures represent total monthly support received from various sources commonly relied upon by older adults in Thailand, including government welfare transfers, family contributions, and personal assets or savings. The high proportion reporting below 5,000 baht underscores persistent socioeconomic vulnerability among this population and highlights the affordability barriers they face in accessing digital health technologies. This passage discusses a study by Jayawardhana et al. (42), regarding economic vulnerabilities in older adults, highlighting the promise of an IoT-based health monitoring system. It builds on earlier research by (43) in aging, technology, and healthcare, illustrating the system's alignment with the goal of universal health coverage outlined in SDG 3.8. The health monitoring system, which consists of a three-tier architecture for data collection, processing, and management, addresses significant challenges in remote health monitoring. The high satisfaction rates with medical devices, particularly smartwatches (93.8%) and blood pressure monitors (81.3%), suggest growing technological acceptance among older adults, which contrasts with earlier assumptions of technological resistance (44). The research assessed a limited version of the system involving elderly users testing wearable sensors and a simple data viewer. Findings do not represent the complete three-tier system, which encompasses emergency alerts and welfare service links. These components remain conceptual and necessitate further technical development, regulatory approval, and significant infrastructure investment before nationwide implementation.

The study of 2,005 older adults in four Thai provinces identified significant socioeconomic and digital inclusion challenges. Compared with the NSO 2024 Survey of Older Person (45) as in Table 1B, our sample over-represents women (76.6% vs. 58.0% nationally) and under-represents those with no formal education (3.0% vs. 67.2% nationally), likely reflecting the community-dwelling, physically mobile nature of our sampling frame. These differences should be considered when generalizing our findings to the broader elderly population in Thailand. With a high female participation rate (76.6%) and a mean age of 68.9 years, many (48.6%) had only primary education, reflecting vulnerability. Economically, 64.2% earned below 5,000 baht monthly, and 15.8% lacked necessary equipment, illustrating barriers to technology access. Notable digital divides were found in areas such as access, skills, and economic status, impacting health equity and technology adoption contrary to SDG 10.3 aims. A concerning 29.9% of participants expressed worries over online fraud, emphasizing the urgent need for enhanced digital literacy programs and user-friendly technology for older adults (46). The analysis revealed a significant digital divide impacting technology adoption, particularly in Khon Kaen Province, where 62.4% have only primary education and 71.9% earn below 5,000 baht. In contrast, Songkhla Province has better educational attainment (40.7% with primary education, 21% holding higher education). These disparities hinder technology acceptance and health service use. Although 92% of residents receive monthly allowances and 77.7% engage in community activities, there's a lack of access to specialized services, with only 48.7% utilizing career development loans. This indicates potential issues in awareness and accessibility of such services (47). Traditional information channels, particularly village news towers (67.2%) and relatives/neighbors (50.9%), are the main sources of information, while digital platforms like the LINE application (32.9%) and Facebook (21.7%) have limited reach.

The study's Technology Acceptance Model (TAM) analysis indicates that older adults show strong acceptance for IoT healthcare technologies, evidenced by high scores in Perceived Usefulness (PU = 4.354), Perceived Ease of Use (PEOU = 4.373), Attitude (AU = 4.352), and Behavioral Intention (BI = 4.259), all exceeding the 4.0 threshold. The reliability coefficients were excellent for PU (*α* = 0.938) and BI (*α* = 0.989), while AU (*α* = 0.664) suggests a need for refinement. Although the AU subscale had lower internal consistency (*α* = 0.664), this falls within the acceptable range for exploratory research by Tavakol et al. (48) and may reflect response clustering at the positive end of the scale, a pattern observed when older adults exhibit acquiescence bias [Hinz, A. et al. (49) and Costello S et al. (50)] and ceiling effects in technology acceptance studies. Studies on technology acceptance in Southeast Asia highlight reported phenomena relating to novel digital health interventions [such as Smith et al. (51) and Kalayou et al. (52)]. However, the findings highlight a critical paradox in Thailand's elderly digital health context: robust individual-level acceptance coexists with pervasive structural barriers that exclude most of the population (e.g., 64.2% inability to afford devices, 48.6% lacking digital literacy, 15.8% without equipment). This suggests that TAM effectively captures motivational factors among current users but may underemphasize systemic exclusion driving low overall adoption rates. Future research should employ larger, more diverse samples—including non-users—and mixed methods (e.g., qualitative interviews) to determine whether elevated scores reflect authentic enthusiasm or methodological artifacts such as response styles. Such approaches could better inform targeted interventions to bridge the user/non-user divide, such as affordability subsidies, literacy training, and user-centered IoT design.

The proposed Social Welfare Rights Enhancement Program for older adults integrates technology through a collaboration of 24 agencies, with budget allocations including 20% from the NBTC and the Ministry of Digital Economy for 5G infrastructure, 15% from the Ministry of Higher Education for digital solutions, and 25% from health and social budgets. The partnership aims for sustainability by mirroring successful international elder care models. However, it faces operational challenges, particularly the exclusion of homebound and bedridden individuals, which limits remote health monitoring access. Context-specific barriers, like vision and memory issues, underscore the necessity for age-friendly technology designs. This study not only addresses local challenges in Thailand but also provides insights valuable to aging societies globally, highlighting demographic and technological challenges similar to those in East Asian and Southeast Asian countries where technological integration in elder care is a policy priority (53–56). The proposed Social Welfare Rights Enhancement Program for older adults addresses critical issues pertinent to emerging economies encountering demographic transitions and resource limitations, emphasizing the necessity of balancing technological advancement with equity for sustainable development.

The health equity impact assessment reveals that while IoT health technologies like smartwatches enhance access, they may worsen disparities for less tech-savvy users. Key limitations include selection bias towards active older adults, neglecting homebound individuals, impacting generalizability. Future research should focus on diverse groups, particularly the homebound and chronically ill. The study advocates for a multi-faceted approach to mitigate barriers, including tailored training, educational programs to build trust, subsidized initiatives for low-income seniors, user-friendly interfaces in local languages, and community peer support. These recommendations align with earlier technology acceptance models by Davis and Venkatesh et al. (57, 58). Comparisons with other similar studies from different countries were also conducted, as shown in Table 5. Future research should focus on inclusive design for the elderly, establish sustainable partnerships, create cost-effective solutions for low-income groups, and provide peer-based technology training in rural settings. Key areas include exploring diverse contexts, intuitive age-friendly interfaces, long-term IoT monitoring effects, and the social-psychological impacts of technology in elder care. The study proposes a framework that includes demographic analysis and technology acceptance, aimed at addressing health equity and disparities while supporting countries in achieving SDGs amid demographic changes.

**Table 5:** Comparison with other similar studies on technology adoption and digital divide among older adults.

Study	Country/ Region	Sample Size	Age Group	Technology Focus	Key Findings	Digital Divide Factors
PIER Study (59)	Thailand (Lampang province)	Vulnerable elderly survey	Elderly	Digital skills and government services	Significant gaps in digital literacy; Education, income, technology access as predictors	Limited device ownership; Internet access barriers, and Education gaps
Jantavongso (60)	Thailand	Literature review	Aging generation	Digital technologies	The digital divide phenomenon prevents older adults from digital opportunities; there is a Need for comprehensive digital literacy programs	Lack of digital literacy; Limited digital opportunities, and COVID-19 acceleration gaps
Choudrie and Vyas (61)	UK (ethnic minority older adults)	Qualitative case study	Older adults	Smart devices (tablets)	The digital divide exists but older adults can be active with proper support; Community engagement crucial	Ethnic minority status; Community support gaps; Digital literacy
Wu & Lim (62)	South Korea	384	60 + years	Smart wearable devices	Performance expectancy, effort expectancy, hedonic motivation significantly affect adoption; Social influence and facilitating conditions important	Digital literacy barriers; Cost concerns, and Age-related barriers
Wang (63)	United States	146	60 + years	Smart wearable systems for health monitoring	Perceived usefulness, compatibility, facilitating conditions, self-reported health status significantly affect intention	Health status variations; Technology compatibility issues
Talukder (64)	China	383	Elderly users	Wearable smartwatch devices	Performance expectancy, effort expectancy, social influence, technology anxiety, resistance to change significant	Technology anxiety; Resistance to change, and Social influence needs
Our study	Thailand	2,005	Mean age: 68.9, 60 + years	IoT health monitoring (smartwatches, blood pressure monitors, glucose meters)	High technology acceptance, 93.8% smartwatch satisfaction	Access (moderate), Skills (significant), Economic (severe), Usage (critical), Geographic (moderate-high)

## 5 Conclusion

This mixed-methods study analyzes social welfare access and digital technology barriers among 2,005 older adults in Thailand, highlighting severe economic conditions and low educational levels that hinder the adoption of digital health solutions. It also includes a 6-month IoT pilot with 80 users. Among the 80 pilot participants who actively used the devices, satisfaction and results of technology acceptance were high overall, suggesting that well-designed, simple IoT health-monitoring tools can be enthusiastically adopted by older adults. However, the positive findings regarding IoT-based approaches for Thailand's aging society cannot be generalized to underrepresented groups, such as homebound or low-income elders. To ensure these innovations are equitable, strategies must include subsidized devices, local language interfaces, peer-led training, and improved rural internet. Without these measures, digital health advancements may worsen health disparities. Future implementation at the national scale should therefore integrate inclusive design and targeted support from the outset to achieve a truly equitable, healthy aging population.

## Data Availability

The original contributions presented in the study are included in the article/Supplementary Material, further inquiries can be directed to the corresponding author/s.
