# Supplementary material for: IoT-based health monitoring and social welfare access for Thailand’s older adults
Source: Front Digit Health. 2026 Apr 7;8:1696118. doi: 10.3389/fdgth.2026.1696118 (PMC13096040; doi:10.3389/fdgth.2026.1696118)
Supplement: Supplementary file 1 [file Datasheet1.docx]

Supplementary Material

# Supplementary Data

# Appendix A

# Research Tool Document

Version 2.0, October 4, 2023

**Questionnaire**

**Guidelines for increasing the potential of access to social welfare to improve the quality of life with digital technology for the Elderly people in Thailand**

## This questionnaire is part of a research project conducted by a group from the Faculty of Science, Khon Kaen University. The project aims to develop guidelines for enhancing access to social welfare and the use of digital technology among the elderly in four regions of Thailand. The objectives of this study are to identify the challenges and obstacles in accessing social welfare services, improve the quality of life for the elderly through digital technology, and examine the roles of government agencies involved in providing these services. Additionally, the study will analyze existing welfare programs to ensure they meet the needs of the elderly and provide recommendations for guidelines that enhance access to social welfare, thereby improving the quality of life and health care for the elderly.

## Incidentally, participation in this questionnaire is voluntary, and respondents' information will be kept confidential for research purposes only. The researcher will not disclose the names of respondents, and there will be no impact on the respondents or their families.

## Section 1: General Information

1. **Respondent**

- Elderly Person

(Please specify both activities you supervise. For each activity, you may select more than one option)

1) Basic Daily Life Activities 2) Work, Travel, and Communication Activities

☐ Bathing/Toileting ☐ Managing money

☐ Dressing ☐ Organizing medication

☐ Feeding ☐ Housekeeping

☐ Getting in and out of bed ☐ Going outside

☐ Walking inside the house ☐ Carrying items

☐ Communication and telephone use

1. **Gender**

☐ Male ☐ Female

1. **Age**

_______ years

1. **Marital Status**

☐ Single ☐ Legally Married ☐ Divorced/Separated

☐ Widowed ☐ Cohabiting (Not Legally Married) ☐ Other: ___________

1. **Education Level**

☐ No formal education ☐ Primary education ☐ Lower secondary education ☐ Upper secondary education / Vocational education ☐ Bachelor’s degree or higher

☐ Other: ___________

1. **Occupation**

☐ Unemployed

☐ Employed

☐ Retired government officer ☐ Business owner ☐ Farmer

☐ General laborer ☐ Landlord ☐ Other: ___________

1. **Income Source**

☐ No income

☐ Has income (Please specify your source of income, listing more than one item if applicable)

☐ 1. Salary/Pension ________ Baht/month

☐ 2.Daily wage/payment per job ________ Baht/month

☐ 3.Support from children ________ Baht/month

☐ 4.Support from relatives ________ Baht/month

☐ 5.Elderly living allowance ________ Baht/month

☐ 6.Income from agriculture/livestock ________ Baht/month

☐ 7.Disability allowance ________ Baht/month

☐ 8.Other ________ Baht/month

Total estimated income ________ Baht/month

1. **Is your income sufficient for your expenses?**

☐ Not sufficient and in debt ☐ Not sufficient but not in debt

☐ Sufficient but no savings ☐ Sufficient and has savings

1. **Expenses for dependents**

☐ No dependents

☐ Has dependents (Please specify number)

☐ Children _____ ☐ Grandchildren _____

☐ Relatives _____ ☐ Others_____

1. **Number of household members**: ________

Living with

☐ Alone ☐ Spouse ☐ Son ☐ Daughter

☐ Son-in-law/Daughter-in-law ☐ Grandchildren ☐ Relatives ☐ Other: ___________

1. **Primary Caregiver**

☐ No caregiver ☐ Spouse ☐ Son ☐ Daughter

☐ Son-in-law/Daughter-in-law ☐ Grandchildren ☐ Relatives ☐ Other: ___________

1. **Who takes care of you when you are sick?**

☐ No one ☐ Spouse ☐ Son ☐ Daughter

☐ Son-in-law/Daughter-in-law ☐ Grandchildren ☐ Relatives ☐ Other: ___________

## Section 2: Additional Information on Housing and Environment of the Elderly

1. **Who is the legal owner of the house where the elderly live?**

☐You are the owner of the house ☐Your spouse is the owner of the house

☐Children ☐Son-in-law/daughter-in-law

☐Relatives ☐House for rent ☐Public areas

☐Other (specify)...................

1. **House/Residence: What equipment is available? Do the elderly use the following equipment?**

| **Equipment** | **Yes** | **No** | **Not**  **known** | **Access** | | **Satisfaction** | | |
| --- | --- | --- | --- | --- | --- | --- | --- | --- |
|  |  |  |  | **Use** | **Not use** | **High** | **Medium** | **Low** |
| Television |  |  |  |  |  |  |  |  |
| Home-based telephone |  |  |  |  |  |  |  |  |
| Computer |  |  |  |  |  |  |  |  |
| Laptop/ Notebook |  |  |  |  |  |  |  |  |
| Ordinary mobile phone for  talking |  |  |  |  |  |  |  |  |
| Smart phones that can use to  access Internet |  |  |  |  |  |  |  |  |
| Blood Pressure Monitoring  device |  |  |  |  |  |  |  |  |
| Smart Watch |  |  |  |  |  |  |  |  |
| Oximeter |  |  |  |  |  |  |  |  |
| Blood Glucose device |  |  |  |  |  |  |  |  |
| Salinity Measuring device |  |  |  |  |  |  |  |  |
| Tablet |  |  |  |  |  |  |  |  |

1. **What system do seniors use to access the internet in their current house or residence?**

☐ The house doesn't use the internet (skip to section 3)

☐ Buy a system from a mobile phone network

☐ Have Wi-Fi at home

☐ At the health service facility

☐ Village Wi-Fi

☐ Others specify.................................................

## Section 3: Health and Health care conditions of the Elderly

1. **Have you ever had a health check?**

☐ Never

☐ Ever (specify)

☐ 1 year ago ☐ 2 years ago ☐ 3 years or more than 3 years ago

1. **Do you have any congenital disease (diagnosed by a medical professional)?**

☐ None ☐ Don't know

☐ Yes (please specify)

1.........................................................  Currently being treated  Not being treated

2.........................................................  Currently being treated  Not being treated

3.........................................................  Currently being treated  Not being treated

4.........................................................  Currently being treated  Not being treated

5.........................................................  Currently being treated  Not being treated

1. **Most of the time, when you are sick, where do you usually go for treatment?**

☐ Didn't do anything ☐ Health stations ☐ Clinics

☐ Government hospitals ☐ Private hospitals ☐ Traditional medicine

☐ Buy medicine at drugstore ☐ Use telemedicine ☐Through the hospital app

☐ Other, specify....................................

1. **How did you go to receive treatment?**

☐ By yourself

☐ There are people who take them. Most of the people who take them to get treatment are;

☐ Children/grandchildren ☐ Spouse ☐ Neighbors

☐ Government officials ☐Other, please specify.......................................

1. **Do you have any medical benefits?**

☐ None because..................................................................................................................................

☐ Yes, what treatment rights do you use?

☐ Health insurance card for seniors (gold card)

☐ Government official card, direct payment card

☐ Social Security Card

☐ Life insurance card

☐ Other(specify) ........................................

1. **Do you have any of the following health problems? If so, which actions do you take to manage them?**

1. Impaired Vision ☐ Never had medical treatment ☐ Previously had medical treatment

☐ Currently being treated

2. Impaired Hearing ☐ Never had medical treatment ☐ Previously had medical treatment

☐ Currently being treated

3. Impaired Walking ☐ Never had medical treatment ☐ Previously had medical treatment

☐ Currently being treated

4. Problems with picking things up

☐ Never had medical treatment ☐ Previously had medical treatment

☐ Currently being treated

5. Difficulty in sitting/lying

☐ Never had medical treatment ☐ Previously had medical treatment

☐ Currently being treated

6. Poor memory/feeling lost/forgetful

☐ Never had medical treatment ☐ Previously had medical treatment

☐ Currently being treated

7. Problems with sleep

☐ Never had medical treatment ☐ Previously had medical treatment

☐ Currently being treated

8. Others, please specify ........................................

## Section 4: Behaviors that elderly exhibit when using digital technology, specifically social media

1. **The device you use**

☐ Your own smartphone ☐ Caregiver's smartphone-style

☐ Your child's smartphone ☐ Laptop ☐ Tablet

☐ Desktop computer (personal computer) ☐ Other (specify)...................

1. **Time spent per usage...................minutes**
2. **Frequency of use**

☐ 1-5 times per day ☐ 5-10 times per day

☐ 11-15 times per day ☐ More than 15 times per day

1. **You use digital technology, specifically social media, to**_______

| **Reasons** | **Use** | **Do not use** | **Frequency** | | |
| --- | --- | --- | --- | --- | --- |
|  |  |  | **Low** | **Medium** | **High** |
| Talk (chat) with children, friends and acquaintances |  |  |  |  |  |
| Entertainment |  |  |  |  |  |
| Read news/posts from friends or others |  |  |  |  |  |
| Buy products |  |  |  |  |  |
| Money transactions |  |  |  |  |  |
| Record own activities, photos or videos |  |  |  |  |  |
| Participate in activities or express opinions in the group |  |  |  |  |  |
| To find routes/ maps when travelling |  |  |  |  |  |
| To check social welfare rights |  |  |  |  |  |
| Use for medical treatment, booking appointments and checking rights in advance |  |  |  |  |  |
| Income from YouTube, TikTok, Facebook page |  |  |  |  |  |

1. **Do you have expenses for using the Internet to access social media? If so, ……………. Baht/month**
2. **The person responsible for your internet usage costs is:**

☐ Yourself ☐ Spouse ☐ Children

☐ Grandchildren ☐ Relatives ☐ other (specify)...................

## Section 5: Impact of Social Welfare on Elderly Quality of Life

1. **Access to social welfare to improve the elderly quality of life**

| **Social Welfare Service** | **Yes** | **No** | **Not**  **known** | **Access** | | **Satisfaction** | | |
| --- | --- | --- | --- | --- | --- | --- | --- | --- |
|  |  |  |  | **Have**  **used** | **Have**  **not used** | **High** | **Medium** | **Low** |
| 1. **Health and medical care** |  |  |  |  |  |  |  |  |
| - Provide advice, consultation or provide knowledge about health and disease for the elderly at least 1 time per year |  |  |  |  |  |  |  |  |
| - Arrange annual health examinations for elderly people at least once a year |  |  |  |  |  |  |  |  |
| - Hospitals have an express lane system for services catering to the elderly and those who are dependent |  |  |  |  |  |  |  |  |
| 1. **Economic Impact** |  |  |  |  |  |  |  |  |
| - Elderly individuals who do not have professional income will receive a living allowance, as specified by law. (They are entitled to a monthly living allowance, which is provided on a step-by-step basis, ranging from 600 to 1,000 baht) |  |  |  |  |  |  |  |  |
| - The agency provides career promotion to the elderly |  |  |  |  |  |  |  |  |
| - Have money to borrow money to pursue a career (Individual 30,000 baht), (Group 100,000 baht) pay in installments without paying interest within 3 years. |  |  |  |  |  |  |  |  |
| - The community supports the establishment of a fund to promote the welfare of the elderly in the community. |  |  |  |  |  |  |  |  |
| - There is a discount service on various items for elderly people, such as tourist attractions or local cultural resources |  |  |  |  |  |  |  |  |
| - Legal children have the right to receive a tax deduction of 30,000 baht per elderly parent for caring for their father or mother |  |  |  |  |  |  |  |  |
| 1. **Social Impact** |  |  |  |  |  |  |  |  |
| - Elderly people can participate in community activities and society, such as being a member of a group (Elderly people or community development groups) |  |  |  |  |  |  |  |  |
| - Elderly people's rights to have an occupation/job placement or vocational training |  |  |  |  |  |  |  |  |
| - Elderly people's rights to education promote education |  |  |  |  |  |  |  |  |
| - Organize social activities such as senior citizens' clubs, passing on the wisdom of the elderly |  |  |  |  |  |  |  |  |
| - Senior citizens have the right to tour government facilities for free. Zoo, historical park, organization, 5 Botanicals |  |  |  |  |  |  |  |  |
| - Elderly people have the right to travel. Reduce fares for the elderly, such as BMTA, BTS Skytrain, subway, MRT Airport Link, buses, trains, boats, and airline tickets as specified. |  |  |  |  |  |  |  |  |
| 1. **Environmental Impact** |  |  |  |  |  |  |  |  |
| - Right to adjust housing conditions, improve and repair the house and adjust the environment appropriately for safety. |  |  |  |  |  |  |  |  |
| - Various agencies must provide places with elevators, stair railings, ramps, parking spaces, and restrooms suitable for the elderly. |  |  |  |  |  |  |  |  |
| - Various agencies organize facilities in the building, vehicles are provided appropriately, such as wheelchair service, etc. |  |  |  |  |  |  |  |  |
| 1. **Aid aspect** |  |  |  |  |  |  |  |  |
| - Elderly people have the right to receive assistance services in difficult situations, help with housing, living, food and clothing |  |  |  |  |  |  |  |  |
| - Elderly people who have been harmed by abuse or being taken advantage of illegally by the law or abandoned, will receive help from government agencies. |  |  |  |  |  |  |  |  |
| - After death, legal children have the right to withdraw money to support funeral expenses for elderly individuals. The amount is 3,000 baht per person, applicable in cases where the elderly person qualifies for a state welfare card. |  |  |  |  |  |  |  |  |

1. **Access channels for information on social welfare to improve the quality of life of the elderly, ensuring that it is conveniently accessible and provides comprehensive information across various fields**

| **Channel** | **Levels of Need** | | | |
| --- | --- | --- | --- | --- |
|  | **Do not want to use** | **A slight desire to use** | **Moderate desire to use** | **High desire to use** |
| 1. Village news tower |  |  |  |  |
| 2. Radio |  |  |  |  |
| 3. Television |  |  |  |  |
| 4. Via Application LINE |  |  |  |  |
| 5. Through the application on the phone |  |  |  |  |
| 1. Facebook |  |  |  |  |
| 7. Search engine/Google |  |  |  |  |
| 8. Chat bot |  |  |  |  |
| 9. Celebrities/people who are trustworthy in society (social influencer) |  |  |  |  |
| 10. Various websites |  |  |  |  |
| 11. YouTube |  |  |  |  |
| 12. Relatives/ Neighbours |  |  |  |  |
| 13. Others, specify…………… |  |  |  |  |

1. **Problems and obstacles in accessing social welfare**

| **Issue/ Problems** | **Do not have Problem** | **Have a problem** | | |
| --- | --- | --- | --- | --- |
|  |  | **Minor** | **Moderate** | **Major** |
| 1. Access to Social Welfare Rights |  |  |  |  |
| 2. Service Speed and Convenience |  |  |  |  |
| 3. Rights Request Channels |  |  |  |  |
| 4. Online Fraud Concerns |  |  |  |  |
| 5. Lack of Digital Technology Equipment |  |  |  |  |
| 6. Digital Device System Compatibility |  |  |  |  |
| 7. Internet/Wi-Fi Coverage |  |  |  |  |
| 8. Digital Technology System Costs |  |  |  |  |

Problems and obstacles in accessing social welfare to improve the quality of life of the elderly, if any

...................................................................................................................................................................................................................................................................................................................................................................................................

For researcher,

Interview form number..................

Interview date..................................

Name of interviewer........................

# Supplementary Figures and Tables


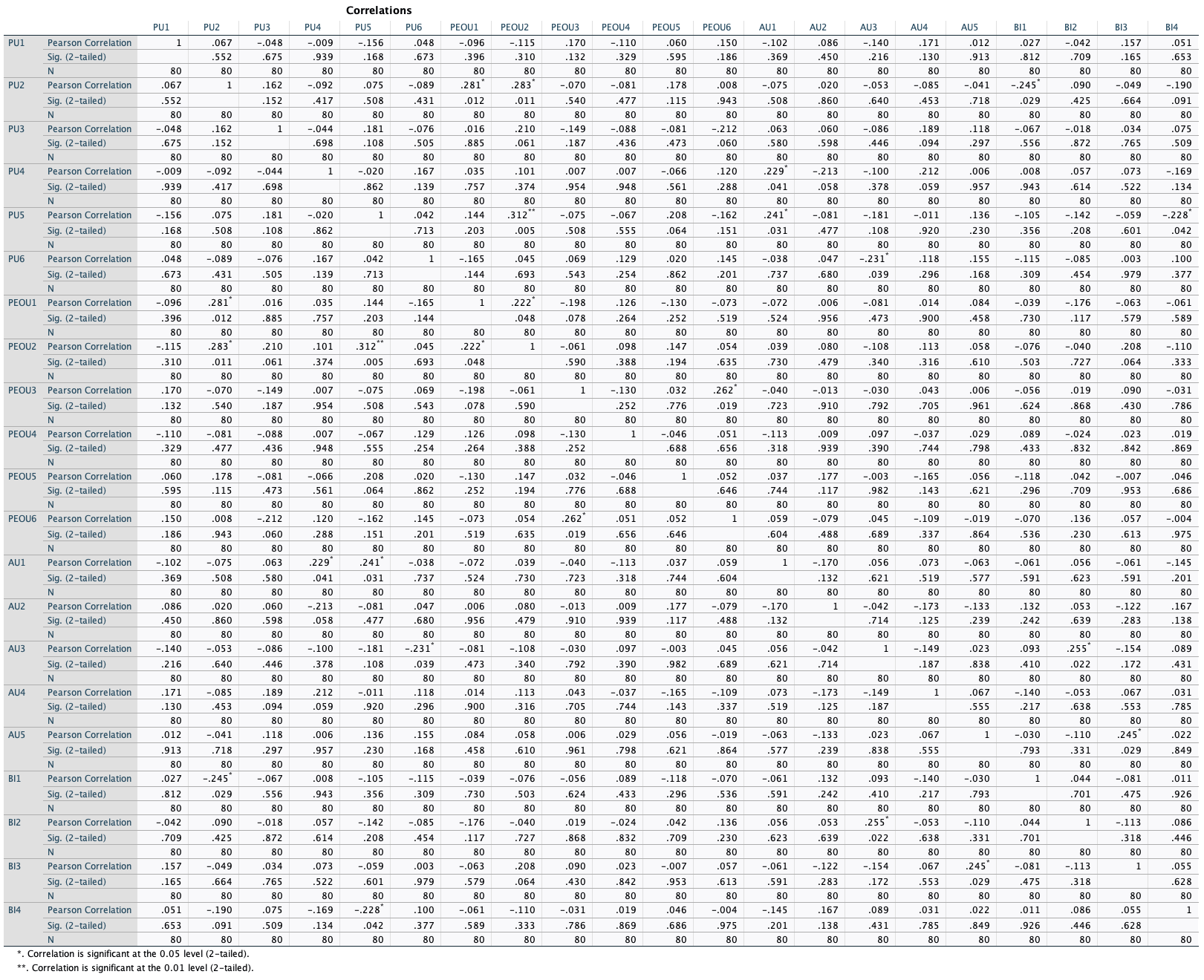


Figure S1. The data analysis of Pearson correlation coefficient.

Table S1. Recognition and utilization of social welfare services among older adults (N = 2,005; Khon Kaen n = 503, Lampang n = 500, Ayutthaya n = 501, Songkhla n = 501). Values are n (%) who reported being aware of and entitled to the service. All percentages are row percentages (within-service distribution across provinces and total).

| Service | Service Description | Khon Kaen | Khon Kaen % | Lampang | Lampang % | Ayutthaya | Ayutthaya % | Songkhla | Songkhla % | Total | Total % |
| --- | --- | --- | --- | --- | --- | --- | --- | --- | --- | --- | --- |
| Healthcare | Health advice  and consultation (annual) | 356 | 70.8 | 379 | 75.8 | 409 | 81.6 | 355 | 70.9 | 1499 | 74.8 |
|  | Annual health  examinations | 417 | 82.9 | 442 | 88.4 | 431 | 86 | 386 | 77 | 1676 | 83.6 |
|  | Express lane hospital  services | 188 | 37.4 | 281 | 56.2 | 246 | 49.1 | 339 | 67.7 | 1054 | 52.6 |
| Economic | Monthly living  allowance (600-1000 baht) | 485 | 96.4 | 482 | 96.4 | 399 | 79.6 | 479 | 95.6 | 1845 | 92 |
|  | Career promotion  services | 254 | 50.5 | 408 | 81.6 | 297 | 59.3 | 333 | 66.5 | 1292 | 64.4 |
|  | Career loan fund | 215 | 42.7 | 301 | 60.2 | 200 | 39.9 | 261 | 52.1 | 977 | 48.7 |
|  | Community welfare  promotion fund | 211 | 41.9 | 367 | 73.4 | 251 | 50.1 | 299 | 59.7 | 1128 | 56.3 |
|  | Discounts for tourist/ cultural attractions | 208 | 41.4 | 396 | 79.2 | 250 | 49.9 | 327 | 65.3 | 1181 | 58.9 |
|  | Tax deduction for  elderly care (30K baht) | 125 | 24.9 | 246 | 49.2 | 163 | 32.5 | 234 | 46.7 | 768 | 38.3 |
| Social | Community and elderly  club participation | 345 | 68.6 | 473 | 94.6 | 346 | 69.1 | 394 | 78.6 | 1558 | 77.7 |
|  | Employment and vocational training rights | 291 | 57.9 | 402 | 80.4 | 278 | 55.5 | 303 | 60.5 | 1274 | 63.5 |
|  | Education rights  (non-formal/  informal) | 255 | 50.7 | 377 | 75.4 | 235 | 46.9 | 310 | 61.9 | 1177 | 58.7 |
|  | Social Activities Organization | 276 | 54.9 | 461 | 92.2 | 305 | 60.9 | 390 | 77.8 | 1432 | 71.4 |
|  | Free Access to Government Sites | 236 | 46.9 | 426 | 85.2 | 265 | 52.9 | 332 | 66.3 | 1259 | 62.8 |
|  | Reduced Transportation Fares | 159 | 31.6 | 412 | 82.4 | 233 | 46.5 | 332 | 66.3 | 1136 | 56.7 |
|  | Housing Condition Adjustment | 208 | 41.4 | 350 | 70 | 254 | 50.7 | 247 | 49.3 | 1059 | 52.8 |
|  | Accessible Facilities | 332 | 66 | 449 | 89.8 | 295 | 58.9 | 360 | 71.9 | 1436 | 71.6 |
|  | Accessible Facility Services | 311 | 61.8 | 460 | 92 | 307 | 61.3 | 362 | 72.3 | 1440 | 71.8 |
|  | Basic Needs Assistance | 249 | 49.5 | 387 | 77.4 | 286 | 57.1 | 265 | 52.9 | 1187 | 59.2 |
|  | Protection from Abuse/  Exploitation | 226 | 44.9 | 360 | 72 | 187 | 37.3 | 253 | 50.5 | 1026 | 51.2 |
|  | Funeral Expense Support | 223 | 44.3 | 387 | 77.4 | 195 | 38.9 | 340 | 67.9 | 1145 | 57.1 |

Table S2. Preferred information channels for learning about social welfare rights and services among older adults (N = 2,005). 1 = No need, 4 = Highly need, values are n (%). All percentages are row percentages (within-channel distribution). Multiple responses were allowed, but most participants selected several channels; “Highly need” reflects the most trusted/effective channels according to respondents.

| Channel | No Need | No Need % | Little Need | Little Need % | Moderately Need | Moderately Need % | Highly Need | Highly Need % |
| --- | --- | --- | --- | --- | --- | --- | --- | --- |
| Village News Tower | 289 | 14.4 | 120 | 6 | 249 | 12.4 | 1347 | 67.2 |
| Radio | 754 | 37.6 | 334 | 16.7 | 293 | 14.6 | 624 | 31.1 |
| Television | 460 | 22.9 | 226 | 11.3 | 415 | 20.7 | 904 | 45.1 |
| LINE Application | 867 | 43.2 | 195 | 9.7 | 284 | 14.2 | 659 | 32.9 |
| Mobile Phone Applications | 1009 | 50.3 | 227 | 11.3 | 275 | 13.7 | 494 | 24.6 |
| Facebook | 1141 | 56.9 | 216 | 10.8 | 213 | 10.6 | 435 | 21.7 |
| Search Engines (Google etc.) | 1404 | 70 | 190 | 9.5 | 153 | 7.6 | 258 | 12.9 |
| Chatbot | 1660 | 83.1 | 134 | 6.7 | 72 | 3.6 | 133 | 6.6 |
| Influencers/  Trusted figures | 1589 | 79.3 | 163 | 8.1 | 100 | 5 | 153 | 7.6 |
| Website | 1496 | 74.6 | 180 | 9 | 139 | 6.9 | 190 | 9.5 |
| YouTube | 1086 | 54.2 | 208 | 10.4 | 253 | 12.6 | 458 | 22.8 |
| Relatives/  Neighbors | 342 | 17.1 | 142 | 7.1 | 500 | 24.9 | 1021 | 50.9 |

Table S3. Problems and obstacles in accessing social welfare services and digital technology among older adults (N = 2,005). Participants rated each issue on a 4-point scale (1 = No problem, 4 = Major problem). Values are n (%). All percentages are row percentages (within-issue distribution).

| Issue/  Problems | No Problem | No Problem % | Minor | Minor % | Moderate | Moderate % | Major | Major % |
| --- | --- | --- | --- | --- | --- | --- | --- | --- |
| Access to Social Welfare Rights | 1785 | 89 | 108 | 5.4 | 59 | 2.9 | 54 | 2.7 |
| Service Speed and Convenience | 1744 | 86.9 | 117 | 5.8 | 78 | 3.9 | 67 | 3.3 |
| Rights Request Channels | 1787 | 89.1 | 92 | 4.6 | 68 | 3.4 | 59 | 2.9 |
| Online Fraud Concerns | 1143 | 57 | 114 | 5.7 | 150 | 7.5 | 599 | 29.9 |
| Lack of Digital Technology Equipment | 1388 | 69.2 | 168 | 8.4 | 133 | 6.6 | 317 | 15.8 |
| Digital Device System Compatibility | 1456 | 72.6 | 161 | 8 | 119 | 5.9 | 270 | 13.5 |
| Internet/WiFi Coverage | 1458 | 72.7 | 139 | 6.9 | 104 | 5.2 | 305 | 15.2 |
| Digital Technology System Costs | 1670 | 83.3 | 108 | 5.4 | 72 | 3.6 | 156 | 7.8 |

Table S4. Social welfare rights and issues Encountered by the Elderly – Interview Records

| Social welfare  rights | Issues that  people encounter | Interview records |
| --- | --- | --- |
| Economic aspect | Increase elderly allowance | “A little more welfare money would help. Right now, we only receive 700 baht per month. We barely spend it before it’s gone. Nowadays, the cost of living is very high.” (S2 Y72)  “What kind of allowance is this? If the elderly allowance were increased, that would be good. Honestly, I don’t know what I want (laughs). It’s things like food—it’s just not enough. What can the government provide? It’s always about money, but the current help isn’t sufficient to cover expenses. I can’t think of anything else right now.” (S5, Y 68)  “It’s not appropriate. It’s just a little oily; it’s not enough.” (K1, Y70)  “I would like to see an increase in the living allowance for the elderly.” (K4, Y 64)  “I would like the elderly allowance to be increased to 3,000 baht per month, giving more support to truly help the elderly. I want it to align with what the government promises. Other than that, I can’t think of anything else. This is all I can think of for now.” (K5, Y65) |
| Social aspect | Did not mention. |  |
| Environmental  aspect | Did not mention. |  |
| Health and  medical care | Did not mention. |  |
| Social work | Distribution and equality for the elderly and disadvantaged, addressing opportunities and necessities. | “There should be a system in place to support elderly individuals who are considered disadvantaged. They should be given genuine opportunities and provided with appropriate care, including necessities like food and shelter, tailored to their condition but without extravagance.” (S1, Y72) |

### Social welfare access, utilization, and needs for older adults' quality of life

Social welfare access varies significantly among services. High-access services include monthly living allowances (92.0% of older individuals without pensions), annual health examinations (83.6%), and community participation (77.7%). In contrast, lower access rates are seen in tax deductions for children caring for older parents (38.3%), career development loans (48.7%), and government assistance for abuse victims (51.2%) (Table S1). Utilization rates align with access, with the most used services being monthly living allowances (86.2%), annual health examinations (83.4%), and health consultation services (82.1%), while the least utilized are government assistance for abuse cases (9.9%), career development loans (13.3%), and funeral expense assistance (19.1%).

Information was primarily accessed by older adults through village news towers (67.2%), relatives and neighbors (50.9%), and television (45.1%), with some using digital platforms like the LINE application (32.9%) and Facebook (21.7%). Key barriers to access included fear of online fraud (29.9%) and lack of digital equipment (15.8%). Senior citizen allowances need enhancement due to rising expenses, with opinions divided on their appropriateness. Many older adults lacked awareness regarding other welfare rights; see Tables S2, S3, and S4.

Table S5. Cronbach's Alpha Coefficient of the test acceptance questionnaire

| **Factor** | **Cronbach's Alpha Coefficient** | |
| --- | --- | --- |
|  | **n=80** | **Numbers of items** |
| Perceived of usefulness: PU | 0.938 | 6 |
| Perceived Ease of Use: PE | 0.836 | 6 |
| Attitude toward using: AU | 0.664 | 5 |
| Behavioral intention: BI | 0.989 | 4 |
| Total acceptance | 0.8567 | 21 |

Table S6. Item-level means, standard deviations, and interpretation of the Technology Acceptance Model (TAM) questionnaire completed by the 80 older adult end-users (n=80), collected via face-to-face interviewer-assisted survey following 6 months of daily device use.

| **Question** | **Mean** | **S.D.** | **Comments** |
| --- | --- | --- | --- |
| **Perceived of Usefulness (PU)** |  |  |  |
| PU01: Using the digital health equipment (KATI watch, WBP202, Contour Plus ELITE, and Gateway MFC-AVA3) helps you track your health data more quickly and easily. | 4.375 | 0.736 | High level of agreement |
| PU02: This equipment enhances the efficiency of providing health services to you. | 4.237 | 0.733 | High level of agreement |
| PU03: The use of this equipment enables you to monitor your health conditions (e.g., blood pressure, blood sugar) more effectively. | 4.425 | 0.632 | High level of agreement |
| PU04: Using these devices improves your ability to manage your health and prevent problems. | 4.312 | 0.739 | High level of agreement |
| PU05: These devices save your time and effort when checking your vital signs. | 4.312 | 0.704 | High level of agreement |
| PU06: You find these devices very useful for looking after your health as an older adult. | 4.462 | 0.673 | High level of agreement |
|  | 4.354 | 0.703 | High level of agreement (Mean >4.0) |
| **Perceived Ease of Use (PE)** |  |  |  |
| PE01: You can learn how to use the KATI smartwatch, blood pressure monitor, and glucometer quickly. | 4.425 | 0.671 | High level of agreement |
| PE02: You can independently control and use these devices without help from others most of the time. | 4.375 | 0.785 | High level of agreement |
| PE03: The steps for measuring and recording your health data with these devices are clear and easy to understand. | 4.354 | 0.657 | High level of agreement |
| PE04: Using this equipment is easier and more convenient than your previous ways of checking blood pressure or blood sugar. | 4.387 | 0.721 | High level of agreement |
| PE05:You can easily become proficient in using this equipment. | 4.351 | 0.713 | High level of agreement |
| PE06: The KATI watch, WBP202, Contour Plus ELITE, and Gateway MFC-AVA3 are user-friendly and uncomplicated. | 4.353 | 0.748 | High level of agreement |
|  | 4.373 | 0.716 | High level of agreement (Mean >4.0) |
| **Attitude towards Using (AU)** |  |  |  |
| AU01: You feel that using these health devices is good for you. | 4.375 | 0.718 | High level of agreement |
| AU02: You like using these devices more than your old methods of checking your health. | 4.375 | 0.753 | High level of agreement |
| AU03: Using these devices has made managing your health more pleasant. | 4.325 | 0.708 | High level of agreement |
| AU04: You think these devices are valuable and suitable for older adults like you. | 4.337 | 0.693 | High level of agreement |
| AU05: You believe that this equipment will become popular in providing health services for the elderly in the future. | 4.351 | 0.695 | High level of agreement |
|  | 4.352 | 0.713 | High level of agreement (Mean >4.0) |
| **Behavioral Intention (BI)** |  |  |  |
| BI01: You plan to continue using this digital health equipment in the future. | 4.225 | 0.795 | High level of agreement |
| BI02: You intend to use this equipment every time you check your health conditions. | 4.225 | 0.711 | High level of agreement |
| BI03: You plan to use these devices a normal part of your daily health routine | 4.225 | 0.795 | High level of agreement |
| BI04: You are satisfied with using this digital health equipment as your personal health monitoring. | 4.362 | 0.716 | High level of agreement |
|  | 4.259 | 0.754 | High level of agreement (Mean >4.0) |
